# The Effects of Computer-Based and Motor-Imagery Training on Scoring Ability in Lacrosse

**DOI:** 10.3389/fpsyg.2020.01588

**Published:** 2020-07-30

**Authors:** Takahiro Hirao, Hiroaki Masaki

**Affiliations:** Faculty of Sport Sciences, Waseda University, Tokorozawa, Japan

**Keywords:** computer-based sport training, shooting performance, Simon task, reversed Simon effect, stimulus–response compatibility

## Abstract

Previous studies have confirmed that the temporal attentional control created by the repetition of stimulus–response compatibility (SRC) tasks was transferred to shooting skills in lacrosse players. In the current study, we investigated whether combining motor imagery training with SRC tasks could enhance the scoring ability of lacrosse players. We grouped 33 male lacrosse players into three groups: an SRC task and motor imagery group (referred as to SRC + Image), an SRC task group, and a control group. Players in the first two groups underwent five sessions of 200 SRC task trials. In addition, the SRC + Image group completed five sessions of motor-imagery training. The control group underwent no training interventions. All three groups performed a lacrosse shooting test and a Simon task before and after training sessions to assess the magnitude of the interference effects of the various types of training they underwent. The results of the Simon task showed that repetition of 1,000 trials was enough to create a short-term representation with the incompatible special mapping being transferred to a dynamic activity like lacrosse shooting. Moreover, a combination of a computer-based Type 2 task and motor-imagery training could effectively increase players’ scoring abilities in a field of large spatial conflict.

## Introduction

Although previous studies for the improvement of lacrosse skills have primarily focused on the physical and biomechanical aspects of training (e.g., Gutowski and Rosene, 2011; Macaulay et al., 2016), comparatively few researchers have shed light on the cognitive dimensions of the sport (e.g., attentional regulation). In lacrosse, shooting in the opposite direction to which the goalie is moving is an effective scoring strategy because the lacrosse goal is narrow (1.83 m × 1.83 m). Effective scorers assess the goalie’s position and movements as quickly as possible and shoot in the opposite direction. However, this is relatively difficult, given that the direction the goalie steps toward is spatially incompatible with the place the shooter should aim. Regardless of the intention of the shooter, their attention is automatically attracted to the goalie’s movement because they automatically process the body orientation of another person (Langton and Bruce, 2000). This automatic allocation of attention is commonly used as a feinting or “fake” technique (Kunde et al., 2011). This automatic attentional mechanism could affect the allocation of the shooter’s attention, resulting in a suboptimal shot execution.

Previous sport-related research has implied that athletes can transfer skills that were obtained *via* executing training on a computer to performance in game situations (Broadbent et al., 2015). Fery and Ponserre (2001) revealed that, through undergoing a computer-based golf-simulated tasks, novice putting skills in golf were improved. Computer-based training is also a helpful intervention for elderly people to train their motor skills, such as gait performance (Pichierri et al., 2012) and postural control (Pluchino et al., 2012; Donath et al., 2016). However, few studies have shed light on the effects of computer-based training on attentional allocation skill, which is required in sport situations. Appropriate control of attention can contribute to better athletic performance (e.g., Vickers, 2011); thus, for competitive athletes, a strategy to modulate the allocation of attention could be important.

In laboratory-based research, the Simon task has been used to investigate the attentional mechanism regarding spatial incompatibility or compatibility between the location of presented stimulus and response hand (Simon, 1969). In a typical Simon task designed for a computer, one of two kinds of stimuli (e.g., a circle colored red or green) is randomly exhibited to the left or the right side of a fixation cross that remains at the center of the monitor. Participants were asked to respond to the red-colored circle with their right hand and to the green-colored circle with their left hand, irrespective of the visible location of the stimulus. When the responding hand matches the location where the stimulus is exhibited, participants’ reaction times (RTs) are shorter than when this was not the case. In other words, the spatial correspondence between stimulus and response affects participants’ RTs. Interference caused by incompatibility between the stimulus location and the required response side is known as the Simon effect, which can be quantified as the difference in RT between incompatible and compatible trials (Proctor and Lu, 1999; Tagliabue et al., 2000, 2002). The unintentional and automatic allocation of attention to the task-irrelevant spatial information (Notebaert et al., 2001) automatically initiates the response preparation corresponding to the stimulus location (Masaki et al., 2000); this causes the Simon effect. On the other hand, the conflict between automatic brain activation associated with the wrong response hand while the spatial information was processed, and the conditional activation regarding the response selection could delay a participant’s response to the incompatible stimulus at the response-selection stage (Masaki et al., 2000). Thus, Simon effects may be caused by processing at both stages, in terms of the perception and of the response selection. This assumption has been supported by previous studies wherein event-related brain potentials were recorded (Valle-Inclán, 1996; Masaki et al., 2000).

Numerous studies have reported that the interference effect of the Simon task is robust (Lu and Proctor, 1995). However, the interference effect of the Simon task may be reversed (i.e., RTs to incompatible stimuli are shorter than those to compatible stimuli) after ample trials of another stimulus–response compatibility (SRC) task where the spatially incompatible reaction to an eccentrically presented stimulus was required (Proctor and Lu, 1999; Tagliabue et al., 2000, 2002). In the Simon task, the stimulus type (e.g., color or letter) and the stimulus location are task-relevant and task-irrelevant, respectively. On the other hand, in the simple SRC task mentioned previously, participants are asked to respond to the opposite side of the location of the stimulus presentation regardless of the stimulus type. In other words, the location of the presented stimulus is task-relevant, whereas for the incompatibility stimulus, the stimulus type is task-irrelevant in the SRC task with an incompatible-spatial mapping. Extended practice of the SRC task in which incompatible-spatial mapping is included may produce a temporal representation of the connection between the incompatible-stimulus location and its response side (Proctor and Lu, 1999; Tagliabue et al., 2000, 2002). Therefore, a reversed Simon effect could be observed after performing ample execution of the SRC task with incompatible-spatial mapping.

A taxonomy proposed by Kornblum (1992) can conceptually distinguish the Simon task and the SRC task with an incompatible-spatial mapping. This taxonomy advocated that individual tasks can be classified into one of eight distinct ensembles considering the dimensional relevance and the dimensional overlap of the stimulus type, the stimulus location, and the response. For the Simon task, the dimension of a piece of task-irrelevant information (i.e., the location of stimulus presentation) is overlapped with the dimension of the response (i.e., a response side), but no overlap exists between the dimensions of a task-relevant piece of information (i.e., a type of stimulus) and response or between the dimensions of task-relevant and irrelevant information. According to this taxonomy, this type of task is designated as a Type 3 ensemble. For the SRC task that was used to reduce the magnitude of the Simon effect in the previous studies, an overlap exists between the task-relevant stimulus (i.e., stimulus location) and response dimensions (i.e., a response side), but such an overlap is absent for the task-irrelevant stimulus (i.e., a type of stimulus) and response dimensions and for the task-relevant and task-irrelevant stimulus dimensions. This type of SRC task is designated as a Type 2 ensemble. Thus, the taxonomy clearly distinguishes between the Simon and SRC tasks with incompatible-spatial mapping.

In a previous study, we clearly showed that the modulation of the spatial attentional allocation induced by 1,800 repetitions of the Type 2 task could transfer to dynamic movements, like the shooting motion in lacrosse (Hirao and Masaki, 2018). In that study, male lacrosse players who acquired a temporal representation for the spatial attentional allocation by executing a total of 1,800 trials of the Type 2 task took more shots in directions opposite to the goalie’s movement.

Although we demonstrated that the repetitive execution of a Type 2 task could have potential as a training tool for proper attentional allocation *vis-à-vis* lacrosse shooting, we found two critical problems with the use of SRC tasks to improve lacrosse shooting. First, the 1,800 repetitions proved a big burden for participants. However, the amount of practice required to induce the reversed Simon effect may be substantially fewer than 1,800 repetitions. In Tagliabue et al. (2000), it was reported that the repetition of 72 trials of a Type 2 task reduced the extent of the Simon effect. To reduce the burden of participants, it is worth testing the effect of fewer repetitions of a Type 2 task on lacrosse shooting performance. Second, the players who underwent a Type 2 task did not see an increase in their scoring ability. They were trained to acquire a strategy for attentional allocation through the Type 2 task, which resulted in an increased number of shots in the opposite direction of the goalie’s movement. However, they did not engage in any physical training during the experiment that aimed in the opposite direction to which the goalie moved. This may explain why they did not enhance their scoring ability; physical training is essential to improve the scoring ability. Thus, it would be fruitful to test whether the execution of SRC tasks, combined with training to improve players’ physical ability to shoot in the opposite direction of a goalie’s movement, can increase their in-game scoring ability.

Motor imagery training can potentially improve players’ physical abilities (Guillot and Collet, 2008). Such training activates brain regions similar to those that are activated by actual movement (Stephan et al., 1995; Gerardin, 2000) and has been proven to be a convenient training tool for athletes (Roure et al., 1999; Robin et al., 2007). Robin et al. (2007) investigated effects of motor-imagery training on service return accuracy in tennis. Their study showed that the players who applied motor-imagery training improved the accuracy of their returns. Although some studies have focused on the auxiliary role of motor-imagery training in executing specific athletic movements, other studies have reported that motor-imagery training can enhance motor learning more generally (Pascual-Leone et al., 1995).

Considering the functional equivalence in the cortical level between a motor execution and imagery, Holmes and Collins (2001) proposed the PETTLEP model. The name of this model was composed of the initial letters of seven elements required for the motor imagery (i.e., physical, environment, task, timing, learning, emotions, and perspective). Although the seven PETTLEP elements are important for the effective motor-imagery training, the physical and environment elements may influence the imagery ability of the player (Anuar et al., 2017). Motor imagery can be divided into participants’ first-person (internal imagery) and third-person (external imagery) perspectives (Holmes and Calmels, 2008) and into visual and kinesthetic sensory modalities (Guillot et al., 2009). Motor-related activations in the brain were more strongly induced by experiencing kinesthetic imagery than visual imagery (Neuper et al., 2005; Guillot et al., 2009). Therefore, motor-imagery training could be an effective tool to improve athletic performance; however, actual training is generally considered to be a more effective means of developing physical ability (Driskell et al., 1994; Gentili et al., 2010; Kraeutner et al., 2020). In the current study, we aimed to develop an effective and convenient training by combining the elements that were proven to be important for the imagery.

We investigated the combined effects of a computer-based SRC task and motor-imagery training on lacrosse players’ shooting performances. We tested whether players’ modulated attentional allocation, acquired *via* less repetition of SRC tasks compared with our previous study (1,800 trials), could transfer to shooting in lacrosse. In previous studies, a repetition of 72 trials was sufficient to reduce the Simon effect immediately after participants executed the spatially incompatible task, 1 day later, and 1 week later (Tagliabue et al., 2000). According to this finding, we hypothesized that a repetition of 1,000 trials would be enough to discern the effects of the SRC task on lacrosse shooting. Moreover, we hypothesized that less repetition of the SRC task (1,000 trials) would be enough to transfer the repetition effects of the SRC task to lacrosse shooting. As a result, participants who trained their attentional allocation and physical action should improve their scoring ability.

## Materials and Methods

### Participants

Participants were recruited from the Waseda University’s male lacrosse team. Thirty-three male lacrosse players participated in the current study. Participants were separated into three groups: the control, SRC, or SRC + image groups (there were 11 participants in each group). One participant in the SRC + image group and three participants in the SRC group were excluded from the analysis because they could not complete an entire schedule of the study due to personal reasons. To minimize pre-existing differences among participants, the three groups were created based on pre-test shooting ability and personal characteristics: age, competitive experience, handedness score, and playing position (i.e., the experimenters divided the participants into three groups so as not to have the statistical difference of any personal characteristics and shooting performance in their pre-test scores). The Edinburgh handedness inventory assessed their hand preferences (Oldfield, 1971). Four out of the 33 participants did not complete the entire experimental schedule for various personal reasons (e.g., injury). The demographic information of the remaining 29 participants is shown in Table 1. A statistical analysis confirmed that there was no difference among groups [age: *F*(2,26) = 0.72, *p* = 0.50; competitive experience: *F*(2,26) = 0.28, *p* = 0.76; handedness score: *F*(2,26) = 1.99, *p* = 0.16]. The ethics committee of Waseda University approved this study (approve number: 2018-155). Prior to participating in the study, all participants were given an explanation about this study. They were also given the option to withdraw from the study whenever they wanted to. Agreement to take part in this study was confirmed by written informed consent from all participants. All participants were paid 1,000 yen per hour during participation.

**TABLE 1:** The characteristics of participants in each group.

	***N***	**Age**	**Competitive experience**	**Edinburgh handedness score**	**Playing position**	
					**AT**	**MD**
SRC + Image	10	20.7 ± 0.9	3.1 ± 1.0	93.3 ± 9.6	3	7
SRC	8	20.8 ± 1.2	2.8 ± 1.2	87.3 ± 15.6	5	3
Control	11	21.3 ± 1.4	3.0 ± 0.9	76.0 ± 28.4	3	8

**The results are presented as mean ± SD, unless stated otherwise. There were no significant differences between the two groups. The number of members were described in the item of playing position (AT, attacker; MD, midfielder)*.*

### Procedure Overview

**FIGURE 1:**
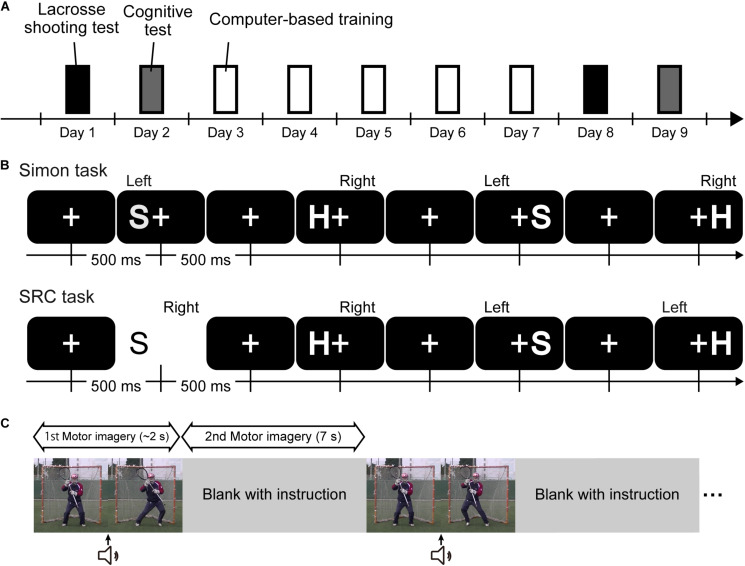
Schematic illustration of the design of the study, cognitive test, and tasks for training. **(A)** Procedure of the current study. **(B)** Trial structure of Simon and SRC task. The correct hand with which to react to each stimulus is depicted just above each stimulus. **(C)** Trial structure of an imagery training. Prior to starting each training, the following instructions were given to participants. “You are standing 8 m in front of the goal, which is guarded by the goalie. Imagine shooting toward the goal after hearing the sound cue as soon as possible (this is the same situation in the shooting test of the pre-test). After the video of the goalie disappears, imagine shooting again (the duration was 7 s). Following the video, imagine shooting 10 trials. Keep in mind the following three things when imaging: Imagine shooting while holding your own lacrosse stick. Imagine shooting from the first-person perspective. Imagine shooting while considering how to move your body to shoot to the opposite direction of the goalie’s movement.”

A schedule of lacrosse shooting tests, cognitive tests, and a training phases were planned over a course of 9 days for all participants. Shooting performance and the magnitude of the Simon effect were assessed before and after the training phase by conducting a lacrosse shooting and a cognitive test, respectively (Figure 1A). On the first day of this study, each participant received an explanation of the experiment and then filled in an informed consent form and questionnaires. Following completion of the questionnaires, the participants executed the lacrosse shooting test. On Day 2, all participants conducted the Simon task. The training program was prepared for the SRC + Imagery and SRC groups from Day 3 to Day 7. The participants in these groups completed their daily assigned training session (nothing was given to the participants in the control group during this training period). On Day 8 and Day 9, the lacrosse shooting, and cognitive test were conducted as the post-test, respectively. However, since we prioritized the schedule of the participants, the time to participate and the experimental environment (e.g., the light and temperature) were not controlled.

The participants performed the computer-based tasks sitting 1 m in front of the laptop monitor with maximum visual angle degrees of 6.1° × 1.9° in both the Simon and Type 2 tasks. For both tasks, the “z” and “/” keys on a keyboard with a layout following Japanese industrial standards were used for measuring the reactions of their left and right index fingers, respectively.

### Shooting Test

In the lacrosse shooting test, a standard-sized lacrosse goal was used (1.83 m × 1.83 m) and each participant was assigned the task of 10 overhand shots while standing 8 m away from the goal, which was guarded by a goalie. The experimenter’s instruction restricting the goalie’s horizontal movement determined the goalie’s initial movement (e.g., step left or step right). Participants were unaware of these instructions. After the experimenter’s initial command, the goalie could move freely to stop the shooter’s attempt. A sound cue provided by the experimenter informed the participants of the beginning of each trial, and both shooter and goalie were instructed to act as soon as possible after hearing the sound cue. The goalies were instructed not to practice typical techniques for guarding the goal (e.g., feints with their eyes or body), because the Simon effect can be affected by processing of the direction of observed gaze or head or body movement (Hietanen, 2002; Ansorge, 2003; Pomianowska et al., 2011). Shooting performances were recorded as video data. Experimenters conceptually divided the goal into a 3 × 3 grid and recorded where the players placed each shot. Shooting performance was evaluated by a performance score, which was calculated by allocating one point for each shot directed opposite to the goalie’s movement and one point for shots that scored (i.e., when participants scored by shooting opposite to the goalie’s movement, they received 2 points). The task in the shooting test was originally developed by our research group to investigate the shooting ability to shoot to the goal guarded by the goalie who initially stepped to the left or right side of the goal. This task was identical to that of our previous study except for the distance from the goal (Hirao and Masaki, 2018).

### Cognitive Test

All participants executed 200 trials of the Simon task to test the degree to which they exhibited the task’s interference effect in the pre- and post-training period. In the Simon task, the visual stimuli were two letters, “H” or “S,” according to the procedure of Proctor and Lu (1999). Participants were required to react by pressing the key using their left or right index finger, ignoring the information in terms of the location of the presented stimulus. The Simon task’s type of visual stimuli was identical to the Type 2 task, which was the task for the training. To avoid confusion between the response rules of the two tasks, eight practice trials in which feedback was provided regarding performance were inserted before the execution of the Simon task in both the pre- and post-test. Participants had to make their responses within 500 ms. Whenever they failed to respond, the Japanese character for “too late” would be shown for 1,000 ms (upper panel in Figure 1B).

### Training Phase

In the SRC + Image group, both the Type 2 task and motor imagery training were used for the intervention. It has been reported that post-training delay was an important factor, which affects the magnitude of the training effect. The retention interval of the motor-imagery training was negatively correlated with the magnitude of the training effect (Driskell et al., 1994). Moreover, the magnitude of the reversed Simon effect after 7 days was larger than executing the SRC task immediately after because of memory consolidation (Tagliabue et al., 2000). To minimize the difference of the post-training delay between two intervention groups, both trainings of the SRC + Image group were conducted on the same day. These two interventions were performed on a laptop. Participants underwent five sessions involving 200 trials of the SRC task (the time span from the first to the fifth training was 9.0 ± 2.6 days). Eight practice trials were inserted prior to starting each training session as well as the cognitive test using the Simon task to assure correct mapping between the stimulus and response. In the Type 2 task, participants were required to react according to the spatial information in terms of the stimulus presentation. Participants were asked to respond using their left finger when characters were presented on the right side and vice versa within 500 ms; otherwise, the Japanese character for “too late” was presented for 1,000 ms (lower panel in Figure 1B).

The motor-imagery training was composed of 10 trials. Following video instruction, participants imagined in response to the goalie’s movement twice in a trial (Figure 1C). At the beginning of each trial, participants were shown a video clip in which a goalie stood at the center of the goal and started to step to the left or right after a sound cue. Participants first imagined shooting in the opposite direction to the goalie’s initial step while watching the clip. The video clip was followed by 7 s of blank screen time. During this period, participants were asked to perform a cognitive rehearsal of shooting again, this time focusing on the kinesthetic aspect of shooting. Participants experienced the motor imagery with a total duration of 7.5 min in the entire training phase. A previous study showed that the approximately 5-min motor imagery had a positive effect on the performance (e.g., Puretz, 1987). Although the volume of the motor training was relatively small compared with the volume of the SRC task, we used the small volume of the motor-imagery training to make our motor-training program convenient and feasible. Participants were required to imagine the shot from the first-person perspective. Participants were also required to imagine the shots while holding their own lacrosse sticks because somatosensory input can impact the quality of motor imagery (Mizuguchi et al., 2015).

The SRC group also used the SRC task, which the SRC + Image group used as training. The number of sessions and trials was also identical to the SRC + Image group except that one participant missed a session due to his schedule (the time span from the first to the fifth training was 9.2 ± 1.8 days). However, in the control group, there was no training intervention. The interval between the pre- and post-test did not differ among the three groups.

### Statistical Analysis

The statistical analyses were conducted using IBM SPSS statistics, version 25 (IBM Corp., Ehningen, Germany). The error rate and RT were subjected to a three-way analysis of variance (ANOVA) with repeated measurements of the variables of test (pre-/post-test) and congruency (congruent/incongruent), and a between-subject variable of group (SRC + Image/SRC/control group). The Simon effect and participants’ scores in the lacrosse shooting test were subjected to a two-way ANOVA with a within-subject variable of test, and between-subject variable of group. When Mauchly’s test violated the assumption of sphericity, the degrees of freedom were adjusted using the Greenhouse–Geisser correction. In pairwise comparisons for *post hoc* tests of the ANOVA, *t*-tests with a multiple-comparison correction using Bonferroni adjustments were used. We used a non-parametric analysis, the Wilcoxon signed-rank test, to evaluate the difference in the number of shots between the pre- and post-test because these data did not show a normal distribution. In all statistical analyses, the significance level for the *p*-value was 0.05. The Cohen’s *d*, $\eta_{\text{p}}^{2}$, and *r*-values were reported as the effect size for the *t*-test, ANOVA, and Wilcoxon signed-rank test (Cohen, 1988; Richardson, 2011).

**FIGURE 2:**
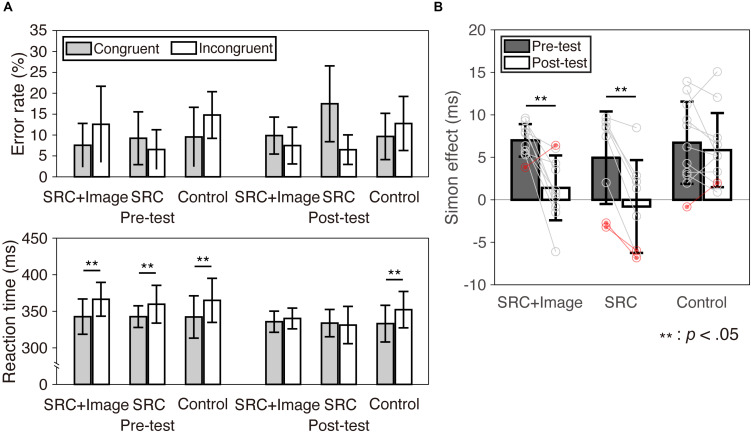
Performance during the cognitive test (Simon task). Error bars represent one standard deviation. **(A)** The error reaction rate and RT are presented. **(B)** The interference effect (Simon effect) of both the pre- and post-test in each group is shown. Individual data were over plotted on the bar graph. The plots in red were individuals who were rejected from the analysis of the lacrosse shooting.

**FIGURE 3:**
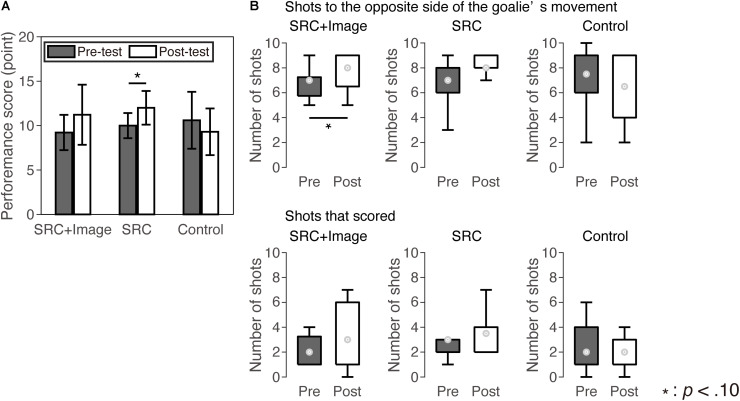
Performance of the shooting test. **(A)** Bar graphs represent mean values of the performance score and error bars represent standard errors. **(B)** The number of shots to the opposite direction of the goalie’s movement (upper panel) and shots that scored (lower panel). Boxplots indicate maximum, minimum, and quartiles of the data. The circles in the boxplots show median values.

## Results

### Cognitive Test (Simon Task)

The results of the Simon task are shown in Table 2 and Figure 2. The magnitudes of the interference effect in both the SRC + Image and SRC groups were significantly reduced, as expected. The control group showed no reduction in the Simon effect (Table 2 and Figure 2B).

**TABLE 2:** Descriptive statistic of the results.

		**Cognitive test (Simon task)**					**Shooting test**
		**Error rate (%)**		**Reaction time (ms)**		**Interference effect (ms)**	**Performance score**
		**C**	**IC**	**C**	**IC**		
SRC + Image	Pre	7.55.2	12.69.1	34324	36623	23.76.0	9.22.0
	Post	9.94.4	7.54.4	33614	34014	4.512.9	11.23.4
SRC	Pre	9.26.3	6.54.7	34315	36026	17.018.8	10.01.4
	Post	17.59.1	6.53.5	33419	33125	−2.718.1	12.01.9
Control	Pre	9.57.1	14.85.6	34229	36530	22.716.0	10.63.2
	Post	9.75.5	12.86.5	33325	35225	19.113.7	9.32.6

**These results were graphically shown in Figures 2, 3. Results are presented as mean ± SD*.*

A three-way ANOVA for the error rate revealed a significant interaction between test and group [*F*(2,26) = 3.66, *p* = 0.040, $\eta_{\text{p}}^{2}$ = 0.22], indicating that the error rate of the SRC group was higher in the post-test than in the pre-test [*t*(7) = 3.83, *p* = 0.006, *d* = 1.06]. On the other hand, there was no significant difference between the pre- and post-test in the other two groups [SRC + Image: *t*(9) = 0.78, *p* = 0.46, *d* = 0.26; control: *t*(10) = 0.66, *p* = 0.052, *d* = 0.18,]. In addition, a significant interaction between compatibility and group was found [*F*(2,26) = 6.64, *p* = 0.005, $\eta_{\text{p}}^{2}$ = 0.34]. The control group responded more accurately in congruent than in incongruent trials [*t*(10) = 2.33, *p* = 0.042, *d* = 0.76]. The SRC group responded more accurately than the control group in incongruent trials [*t*(17) = 3.11, *p* = 0.019, *d* = 1.45]. An interaction between compatibility and test was also significant [*F*(1,26) = 21.0, *p* < 0.001, $\eta_{\text{p}}^{2}$ = 0.45]. Differences between the pre- and post-test were found in both congruent and incongruent trials [*t*(28) = 2.45, *p* = 0.021, *d* = 0.47, *t*(28) = 2.44, *p* = 0.021, *d* = 0.38, respectively] (Table 2 and upper panel in Figure 2A).

A three-way ANOVA for RT revealed that the main effect of compatibility was significant [*F*(1,26) = 32.1, *p* < 0.001, $\eta_{\text{p}}^{2}$ = 0.55], and this was also the case for test [*F*(1,26) = 22.5, *p* < 0.001, $\eta_{\text{p}}^{2}$ = 0.46]. An interaction between test and compatibility was also significant [*F*(1,26) = 39.0, *p* < 0.001, $\eta_{\text{p}}^{2}$ = 0.60]. In addition, an interaction among group, time, and compatibility were significant [*F*(1,26) = 5.91, *p* = 0.008, $\eta_{\text{p}}^{2}$ = 0.31]. *Post hoc* tests confirmed that longer RTs in incongruent than in congruent trials were observed in the pre-test for all three groups [SRC + Image: *t*(9) = 12.6, *d* = 1.01, *p* < 0.001; SRC: *t*(7) = 2.56, *p* = 0.037, *d* = 0.81; control: *t*(10) = 4.69, *p* = 0.001, *d* = 0.77], but the differences in RT diminished for both the SRC + Image and SRC groups in the post-test [SRC + Image: *t*(9) = 1.10, *p* = 0.30, *d* = 0.31; SRC: *t*(7) = 0.42, *p* = 0.69, *d* = 0.12]. For the control group, the difference in RT remained in the post-test [*t*(10) = 4.63, *p* = 0.001, *d* = 0.77] (Table 2 and lower panel in Figure 2A).

A two-way ANOVA for the interference effect in the Simon task revealed that a main effect of the test reached significance [*F*(1,26) = 39.0, *p* < 0.001, $\eta_{\text{p}}^{2}$ = 0.60]. In addition, a significant interaction between test and group was obtained [*F*(2,26) = 5.91, *p* = 0.008, $\eta_{\text{p}}^{2}$ = 0.31] (Table 2 and Figure 2B). The Simon interference effect was attenuated for both the SRC + Image and SRC group [*t*(9) = 4.08, *p* = 0.002, *d* = 1.92, *t*(7) = 5.69 *p* = 0.001, *d* = 1.07, respectively], whereas the control group did not show any attenuation [*t*(10) = 1.06 *p* = 0.32, *d* = 0.24].

### Performance

One participant did not show a reversed Simon effect; nevertheless, he repeated the Type 2 task. Three participants had a reversed Simon effect in the pre-test. We removed these participants from further analysis.

Table 2 and Figure 3A shows the performance scores of participants during the lacrosse shooting test in the pre- and post-test in each group. A two-way ANOVA for the performance score revealed a significant interaction between test and group [*F*(2,22) = 3.59, *p* = 0.045, $\eta_{\text{p}}^{2}$ = 25]. The *post hoc t*-tests showed that only the SRC group tended to increase shooting scores in the post-test compared to the pre-test [SRC + Image: *t*(8) = 1.85, *p* = 0.10, *d* = 0.72.; SRC: *t*(5) = 2.34, *p* = 0.067, *d* = 1.20; control: *t*(9) = 1.27, *p* = 0.24, *d* = 0.44].

We used the performance scores to analyze and assess participants’ abilities to shoot in the direction opposite to the goalie’s movement and to score. To conduct a detailed analysis to compare pre- and post-test performance, we applied a Wilcoxon test to the number of shots opposite to the direction of the goalie’s movement and the number of scoring shots. The SRC + Image group tended to increase the number of shots in the opposite direction to the goalie’s movement from the pre- to post-test (*Z* = 1.73, *p* = 0.084, *r* = 0.58). However, this was not the case for the other two groups (SRC: *Z* = 1.63, *p* = 0.10, *r* = 0.67; control: *Z* = 0.72, *p* = 0.47, *r* = 0.23] (Figure 3B, upper panel). On the other hand, the number of scoring shots did not differ between the two tests for all three groups (SRC + Image: *Z* = 1.13, *p* = 0.26, *r* = 0.38; SRC: *Z* = 1.51, *p* = 0.13, *r* = 0.62; control: *Z* = 0.89, *p* = 0.37, *r* = 0.28] (Figure 3B, lower panel).

**FIGURE 4:**
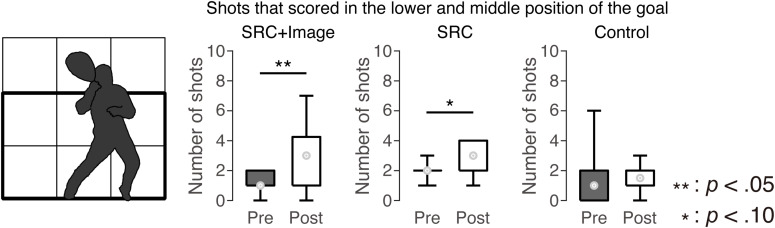
The number of shots that scored in the lower and middle position of the goal. Boxplots indicate maximum, minimum, and quartiles of the data. The circles in the boxplots show median values.

Experimenters recorded where the players shot in the conceptual 3 × 3 goal grid. Little horizontal movement of the goalie was observed in the upper area of the goal; therefore, we focused on shots into the lower and middle sections of the goal (Figure 4, left panel) to investigate the difference in scoring shots between the pre- and post-test. The Wilcoxon test revealed an improvement in scoring ability both for the SRC + Image group (*Z* = 2.05, *p* = 0.041, *r* = 0.68) and SRC group (*Z* = 1.89, *p* = 0.059, *r* = 0.77). However, the control group did not show a difference between the pre- and post-test (*Z* = 0.29, *p* = 0.77, *r* = 0.09).

## Discussion

This study investigated the effects of combining a computer-based SRC task with motor-imagery training on lacrosse shooting performance. We also investigated the transfer of modulated attentional allocation acquired through less repetition of an SRC task to lacrosse shooting.

In the cognitive test of the Simon task, the interference effect (i.e., the difference in RT between congruent and incongruent trials) was reduced from the pre- to post-test for the SRC + Image (24 ± 6 ms for pre-test and 4 ± 13 ms for post-test) and SRC group (17 ± 19 ms for pre-test and −3 ± 18 ms for post-test), although no reduction was found for the control group (23 ± 16 ms for pre-test and 19 ± 14 ms for post-test). The age of the participants might cause this incomplete-reversed Simon effect. Previous studies have implied that the magnitude of the Simon effect can be ascribed to the age group. Participants aged 5–8 years have demonstrated a complete reversal of the Simon effect (Tagliabue et al., 2000), although participants aged 19–25 years have demonstrated a fading magnitude of the Simon effect (Tagliabue et al., 2000; Hirao and Masaki, 2018). It is reasonable to assume that the Simon effect in young adults may not be reversed because a representation of a strong link between stimuli and response locations was fully consolidated. It is plausible that participants who were recruited in the present study (aged 19–24 years) had already developed this firm representation, which has precluded them from reaching the complete-reversed Simon effect. However, it should be emphasized that the participants who executed 1,000 trials of a Type 2 task showed a reduction of the Simon effect. Tagliabue et al. (2000) reported that 72 trials of the SRC task with incompatible-spatial mapping decreased the amount of the Simon effect 1 week after executing the Type 2 task. These studies suggest that ample repetition of the SRC task is not always necessary to make a short-term representation of the incompatible-spatial mapping, which reduces the interference effect. There were three participants (two for the SRC group and one for the control group) who exhibited a reversed Simon effect even before the training intervention; therefore, we excluded these participants from the analyses. The result was likely due to the instruction given for the SRC task. The Simon effect was reversed only when the instruction associated with incompatible-spatial mapping was presented in an SRC task (Theeuwes et al., 2014). Prior to participating in this study, all participants were given the explanation in terms of the training. This explanation for the study could affect the performance in the pre-test of the Simon task. Another explanation could be the individual difference of the top-down processing for the stimulus. In the previous study, the reversed interference effect could be observed by changing the stimulus type (Müsseler et al., 2009). The individual difference of the cognition about the stimulus may influence the magnitude of the interference effect in the Simon task. Although one participant did not show a reversed Simon effect, he repeated the Type 2 task; the result may be caused by the individual difference of the ability of the stimulus cognition.

More interestingly, the SRC group tended to improve their performance scores through the Type 2 task. Performance scores simultaneously assessed shooters’ abilities to score and shoot in the opposite direction of the goalie’s initial step. Although these scores reflected comprehensive skills, scoring and shooting abilities might not be independent of one another because it could be an effective scoring strategy to shoot in the opposite direction of the goalie’s movement. Therefore, it was ambiguous that ability was improved. However, considering that the attentional modulation was induced by the repetition of the SRC task transferred to the lacrosse shooting skill in the previous study (Hirao and Masaki, 2018), it may be plausible that the SRC group improved their attentional allocation.

To investigate the detailed effects of training, we applied non-parametric tests to compare the number of scoring shots and shots placed in the direction opposite to the goalie’s movement in the pre- and post-test because these data did not entirely show a normal distribution. The analysis revealed that the SRC + Image group marginally improved their ability to shoot toward the spot from which the goalie was moving away. Given that the Simon effect was only reduced in the two groups that engaged in repetitions of the Type 2 task, the incompatible-spatial mapping, which was created by the Type 2 task, would transfer to lacrosse shooting performance, which was consistent with our previous finding (Hirao and Masaki, 2018). Another possibility for this result is that the motor-imagery training could modulate the shooter’s attention. In the instruction for the motor-imagery training, participants were asked to imagine to shoot in the opposite direction of the goalie’s movement. There was a possibility that the instruction for the motor imagery and the motor-imagery training by using this instruction induced the change of the attentional allocation of the participants. This interpretation might be supported by previous study in which the representation between the stimulus location and its response to the opposite side of the stimulus presentation could be easily created, like receiving the instruction of the SRC task with incompatible-spatial mapping (Theeuwes et al., 2014).

Contrary to our hypothesis, the SRC + Image group did not increase their number of scoring shots. One possible explanation for this is the interval between the last motor-imagery training and the shooting test. In the current study, the SRC + Image and SRC groups conducted the post-test lacrosse shooting test approximately 1 week after the last training (the SRC + Image: 6.4 ± 2.7 and SRC group: 7.5 ± 2.1). The post-test was conducted a significant length of time after the intervention, so the effect of the motor-imagery training may have disappeared. Driskell et al. (1994) revealed a negative correlation between the effect of motor-imagery training and post-training delay. Therefore, the effects of motor-imagery training are more apparent when measured after a shorter delay. Another possible explanation for this result is the type of task, which is proposed as an important element for the motor imagery by the PETTLEP model. The lacrosse shooting might be too complicated to imagine in the short duration of this study (a total of 9 s was given to the participant in each trial). The review of the motor imagery implied that the task difficulty was inversely related to the duration of the motor imagery (Decety, 1996). The duration of the imagery training in this study may be not enough to have the positive effect. Lastly, this result may also be attributed to the individual differences in motor-imagery ability. It is difficult to improve sport performance by conducting motor-imagery training with participants who have poor imagery ability (Robin et al., 2007). Thus, there is a possibility that participants in the SRC + Image group were not as affected by motor imagery to improve their scoring ability due to their own limited imagery ability.

However, the SRC + Image group did improve some of the skills necessary to score. Statistical analyses of shots to the middle and lower parts of the goal showed that the SRC + Image group improved their scoring ability from the pre-test to post-test. Because processing motion is associated with a visual attentional shift (Treue and Maunsell, 1996), the conflict between the goalie’s movement and the shooter’s aiming point might have been stronger in the shots to both the middle and lower parts of the goal. Only shots to the area in which a stronger spatial conflict existed could be influenced by the training. Another possible reason why SRC + Image group increased scores in the middle and lower parts of the goal is the difference of the difficulty to score among the shots to upper, middle, and lower parts of the goal. However, since the goalie held his cross while the head of the cross was up (see the goalie in the instruction video for the imagery training in Figure 1C), the shooter found it difficult to aim for the shots in the upper parts of the goal.

In the current study, the effects of computer-based and motor-imagery training on lacrosse shooting might be best observed in shots to the middle and lower positions of the goal. This result implied the positive synergistic effects of computer-based SRC task and motor-imagery training on their scoring skill. The repetition of the SRC task in which incompatible-spatial mapping is included, may produce a temporal representation of the connection between the stimulus location and its response to the opposite side of the stimulus location (Proctor and Lu, 1999; Tagliabue et al., 2000, 2002). Moreover, the temporal representation could transfer to the attentional allocation of the shooters in the lacrosse (Hirao and Masaki, 2018). According to these findings, the SRC task could contribute the attentional shift of the shooters in lacrosse. This interpretation might be supported by the results of the performance score in the SRC group.

In shots to the middle and lower parts of the goal, improvement of scoring skill was found for the SRC + Image group, but not for the SRC group, implying a beneficial effect of the motor-imagery training on performance. In our study, instructions of motor imagery included kinesthetic aspects as well as visual processing. Considering that the kinesthetic motor imagery activates the motor-related brain area (Neuper et al., 2005; Guillot et al., 2009; Ruffino et al., 2017), it is plausible that our motor-imagery training enhanced the motor skill to shoot at the advantageous spot of the goal. Another possible explanation for the beneficial effect is that observing the goalie’s movement in the video used for the motor-imagery training improved the scoring skill. A previous study suggested that the video-based simulation may be an effective training to improve the perceptual-cognitive skills (Broadbent et al., 2015). Therefore, the participants might have also improved their perceptual skills associated with determination of the goalie’s movements, resulting in enhancement of the scoring ability. It can be concluded that the shooters may acquire the short-term representation with the incompatible-spatial mapping by repeated execution of the SRC task and the motor and/or cognitive-perceived skills by the motor-imagery training. However, it should be noted that it was uncertain whether our results in the SRC + Image training were attributed to the summation or synergistic effect of the two trainings; the combination of the SRC task and the motor-imagery training could contribute to the improvement of the scoring skill.

This study is not without its limitations. One of these was the method for the group assignment. The participants were divided into three groups depending on their personal characteristics and their pre-test results. The participants were not randomly assigned to each group. Moreover, the cognitive test (i.e., Simon task) was executed between the training session and lacrosse shooting test. The small number of trials in the Simon task could impact the short-term representation of the spatial mapping, which was created by the repetition of the SRC task (Tagliabue et al., 2000). In the Simon task, the participants were required to respond to the stimulus, which was presented at the same side of the response. The execution of the Simon task likely impaired the short-term representation that was created in the training session. Furthermore, the experimental environment in this study was far from the game situation in lacrosse. For example, in the current study, the goalie’s initial movement was restricted in stepping to the right or left side of the goal. Therefore, we could not clearly state whether the results in this study could transfer to the lacrosse performance in the game situation. In addition to this, the limitation was the motor-imagery training. The individual difference of an imagery ability and the evaluation of the executed motor imagery were not measured. The motor-imagery ability measured by the questionnaire could influence the benefit from execution of the motor-imagery training (Robin et al., 2007). Although we instructed the participants to imagine the lacrosse shooting from the first-person perspective with a kinesthetic sensation, this manipulation was not evaluated after imaging. The participants had an imagery perspective preference and could switch the type of perspectives in their imagery (e.g., Roberts et al., 2010). Moreover, the PETTLEP elements of timing, learning, and emotions were not controlled. The final limitation was the training volume. There was a possibility that the training volume may not be enough to get the training effect on the lacrosse performance. Although we used a small volume of the training to develop the convenient training program, the training volume may have been too small. Moreover, the training volume was different between the two intervention groups. This difference could influence the result of the shooting.

## Conclusion

The results of the Simon task showed that repetition of 1,000 trials was enough to create a short-term representation with the incompatible special mapping and transfer to a dynamic performance like lacrosse shooting. We replicated previous findings that repetition of a Type 2 task modulates the attentional allocation in lacrosse shooting. Moreover, it was shown that a combination of computer-based Type 2 and motor-imagery training was an effective way of increasing the players’ scoring ability by comparing their shooting performances among three groups. However, it should be noted that the effect of the combination of the two training interventions at an individual level was unclear because the effect of motor-imagery training on individuals depends on their own motor-imagery ability.

## Data Availability Statement

The datasets generated for this study are available on request to the corresponding author.

## Ethics Statement

The studies involving human participants were reviewed and approved by the ethics committee of Waseda University. The patients/participants provided their written informed consent to participate in this study.

## Author Contributions

TH and HM contributed to the conception and design of the study. TH acquired and analyzed data and drafted the manuscript. HM critically revised the manuscript. Both authors approved the version of the manuscript to be published.

## Conflict of Interest

The authors declare that the research was conducted in the absence of any commercial or financial relationships that could be construed as a potential conflict of interest.
